# 95262 Making telehealth accessible for patients who are visually impaired: A scoping review

**DOI:** 10.1017/cts.2021.533

**Published:** 2021-03-30

**Authors:** Stephanie Zawada

**Affiliations:** Mayo Clinic Graduate School of Biomedical Sciences

## Abstract

ABSTRACT IMPACT: By outlining telehealth access disparities in the vision-impaired population, this scoping review has identified a set of effective and clinically appropriate implementation strategies and interventions for improving the technical, provider-level, and system-level accessibility of telehealth for vision-impaired patients. OBJECTIVES/GOALS: Evidence-based recommendations that ensure telehealth access for vision-impaired patients are critical to reducing health disparities. This review identifies, evaluates, and proposes strategies for public and private sector stakeholders to increase telehealth access for vision-impaired patients during the pandemic and beyond. METHODS/STUDY POPULATION: This scoping review included five steps: 1) the implementation of an iterative search strategy using relevant keywords to query 4 electronic databases (PubMed, Cochrane, Google Scholar, and Europe PMC) for relevant articles, 2) the application of a set of inclusion criteria to filter database results for article evaluation, 3) a quality assessment of the articles retained, 4) the extraction and summary of data from each assessed article, and 5) a narrative synthesis of the qualitative literature reviewed. RESULTS/ANTICIPATED RESULTS: To date, 21 articles that fit the inclusion criteria, published between 2006 and 2020, have been identified. To ensure the most robust collection of existing literature is aggregated, the iterative search strategy and inclusion criteria sorting process will be underway until December 20. The assessment of articles, and extraction and summary of data contained within said articles, will be finalized on January 20. The narrative synthesis will be complete on February 1. The poster and abstract will be complete by February 20. DISCUSSION/SIGNIFICANCE OF FINDINGS: Future research should examine outcomes associated with the implementation of accessible telehealth programs to identify remaining barriers. To improve outcomes for vision-impaired patients, policymakers, providers, payers, and industry must collaborate to promote accessibility in telehealth design and implementation.

